# Deep Eutectic Solvent Coated Cerium Oxide Nanoparticles Based Polysulfone Membrane to Mitigate Environmental Toxicology

**DOI:** 10.3390/molecules28207162

**Published:** 2023-10-19

**Authors:** Saif-ur-Rehman, Muhammad Shozab Mehdi, Muhammad Fakhar-e-Alam, Muhammad Asif, Javed Rehman, Razan A. Alshgari, Muddasar Jamal, Shafiq Uz Zaman, Muhammad Umar, Sikander Rafiq, Nawshad Muhammad, Junaid bin Fawad, Saiful Arifin Shafiee

**Affiliations:** 1 Department of Chemical Engineering, COMSATS University Islamabad, Lahore Campus, Defence Road, Off Raiwind Road, Lahore 54000, Punjab, Pakistan; muddasar_18002825@utp.edu.my (M.J.); gourmani5@yahoo.com (J.b.F.); 2 Interdisciplinary Research Center in Biomedical Materials, COMSATS University Islamabad, Lahore Campus, Defence Road, Off Raiwind Road, Lahore 54000, Punjab, Pakistan; 3 Department of Chemical Engineering, Ghulam Ishaq Khan Institute of Engineering Sciences and Technology, Topi 23460, Khyber Pakhtunkhwa, Pakistan; shafiqzaman029@gmail.com (S.U.Z.); umardurrani07@gmail.com (M.U.); 4 Department of Physics, GC University Faisalabad, Faisalabad 38000, Punjab, Pakistan; fakharphy@gmail.com (M.F.-e.-A.); asif3220286@gmail.com (M.A.); 5 State Key Laboratory of Metastable Materials Science and Technology, School of Materials Science and Engineering, Yanshan University, Qinhuangdao 066004, China; javed_rehman15@yahoo.com; 6 Department of Chemistry, Kulliyyah of Science, International Islamic University, Malaysia, Jalan Sultan Ahmad Shah, Kuantan 25200, Pahang, Malaysia; sabs@iium.edu.my; 7 MEU Research Unit, Middle East University, Amman 541350, Jordan; 8 Chemistry Department, College of Science, King Saud University, Riyadh 11451, Saudi Arabia; ralshgari@ksu.edu.sa; 9 Department of Chemical Engineering, Universiti Teknologi PETRONAS, Bandar Seri Iskandar 32610, Perak, Malaysia; 10 Department of Chemical, Polymer and Composite Materials Engineering, University of Engineering and Technology Lahore, New Campus, Lahore 39161, Punjab, Pakistan; sikander@uet.edu.pk; 11 Department of Dental Materials, Institute of Basic Medical Sciences, Khyber Medical University, Peshawar 25100, Khyber Pakhtunkhwa, Pakistan; nawshad.ibms@kmu.edu.pk

**Keywords:** interface engineering, deep eutectic solvents, ceria nanoparticles, membranes, gas separation

## Abstract

In this study, ceria nanoparticles (NPs) and deep eutectic solvent (DES) were synthesized, and the ceria-NP’s surfaces were modified by DES to form DES-ceria NP filler to develop mixed matrix membranes (MMMs). For the sake of interface engineering, MMMs of 2%, 4%, 6% and 8% filler loadings were fabricated using solution casting technique. The characterizations of SEM, FTIR and TGA of synthesized membranes were performed. SEM represented the surface and cross-sectional morphology of membranes, which indicated that the filler is uniformly dispersed in the polysulfone. FTIR was used to analyze the interaction between the filler and support, which showed there was no reaction between the polymer and DES-ceria NPs as all the peaks were consistent, and TGA provided the variation in the membrane materials with respect to temperature, which categorized all of the membranes as very stable and showed that the trend of stability increases with respect to DES-ceria NPs filler loading. For the evaluation of efficiency of the MMMs, the gas permeation was tested. The permeability of CO_2_ was improved in comparison with the pristine Polysulfone (PSF) membrane and enhanced selectivities of 35.43 (α_CO2/CH4_) and 39.3 (α_CO2/N2_) were found. Hence, the DES-ceria NP-based MMMs proved useful in mitigating CO_2_ from a gaseous mixture.

## 1. Introduction

CO_2_ is the major impurity found in natural gas, which is a major contributor to methane and is an important source of renewable energy [1]. The CO_2_ in natural gas causes the corrosion of pipelines and reduces its calorific value [2]. Also, as it is a greenhouse gas, it results in a thermal effect on the environment by absorbing radiation which gives rise to global warming. Concerning the sources of CO_2_ emissions, power generation is the major CO_2_ discharge source, which is due to the combustion of natural gas and coal or other fossil fuels for the production of electricity [3]. The burning of fuels in vehicles on a domestic level, as well as in other industries, are further sources of CO_2_ emissions. Hence, to reduce greenhouse gas emissions and global warming, to improve economic efficiency, and to provide a sustainable source of energy, it has become necessary to separate CO_2_ from natural gas [4]. As well as CO_2_/CH_4_ separation, CO_2_/N_2_ separation is also necessary as it improves the cleanliness of the atmosphere and reduces the percentage of CO_2_ in the environment [5].

Membrane technology has attracted researchers due to its attributes of having a small footprint, being easy to operate and having a low energy consumption in separating CO_2_ [6]. Membrane-based materials have great significance in this field [7] and organic polymeric membranes are leading the market, as compared to other two-dimensional materials [8] and organics [9,10,11]. The reasons for this are the low cost, easy processing, and high mechanical strength of the polymers [12,13]. The major problem faced in this field is overcoming the permeability and selectivity trade-off as explained by Rosebeson [14]. The incorporation of novel advanced fillers on polymeric support to synthesize membranes may provide an effective solution to optimize the perm-selectivity trade-off [15,16].

The synthesis of mixed matrix membranes (MMMs) has some drawbacks, like fewer interactions between filler particles and polymers, the non-uniform dispersion of the filler on polymer, and chances of a reaction between the filler and polymers [17,18]. The interface morphology of MMMs is also a significant factor in separation via membrane and is controlled by novel supervenient materials as filler. Covalent organic frameworks (COFs), metal organic frameworks (MOFs), metal oxides, alumina, silica, zeolites, nanoparticles, nanoghraphenes, and ceramics are the materials that are being used as filler for CO_2_ separation in different separation techniques, as well as in the fabrication of MMMs [19,20,21]. Sometimes inimical problems occur due to an acidic or humid environment, which can become the cause of interface incompatibility between the filler and polymer [22]. The specification of the filler plays a key role in overcoming this type of problem, and desirable features can be imparted to the membranes by introducing specific fillers in the membrane matrix [23,24].

Recent studies have employed nanoparticles as the filler in mixed matrix membranes. Hasebe et al. [25] has applied silica nanoparticles in polymeric membranes to separate CO_2_ as a way to overcome the Robeson upper bound limit, which resulted in a cost-effective solution for high performance CO_2_ separation in the form of permeability and selectivity (CO_2_/N_2_) on large scale. Raouf et al. [26] have used graphene hydroxyl nanoparticles and polysulfone/polyethylene glycol to synthesize MMMs and obtained a permeability in the range of 15.9–28.2 Barrer and a selectivity (CO_2_/CH_4_) range of 12.23–12.81 at 2 bar pressure with a maximum of 22.39 at 8 bar pressure. Sainath et al. [27] grew ZIF-67 NPs on PSF/GO hollow fiber membranes (HFMs) to increase the CO_2_ removal from natural gas and reported selectivities of 44.94 ± 3.00 and 22.38 ± 0.30 for mixed and pure, respectively. Ruhaimi et al. [28] synthesized spherical CeO_2_ nanoparticles to apply in egg-shell membranes as a bio-template for the high efficiency of CO_2_ adsorption. Farashi et al. [29] has improved CO_2_ separation from CH_4_ using a Pebax-1657 membrane with the addition of alumina (Al_2_O_3_) NPs in the membrane matrix and reported CO_2_ permeability as 159.27 Barrer and selectivity as 24.73 at 8% Al_2_O_3_ NP loadings by weight with different characterizations of membrane. And Xu et al. [30] reported the high NPs loadings in MMMs with the help of chemical bridging-crosslinking for the high performance separation of CO_2_ and found the permeation of CO_2_ and selectivity (CO_2_/N_2_) to be 1295 GPU and 91 at 0.3 MPa, respectively.

In this study, the purpose was to mitigate the amount of CO2 from the gaseous mixture to reduce the toxicity of the gases, like natural gas, flue gases etc. A lot of materials have been used for this purpose, so there was the need to make a novel combination of the compounds that could have the potential to give better outcomes for this purpose. Following this purpose, DES immobilized cerium oxide (CeO_2_) is used in MMMs for CO_2_ separation. DESs are popular candidates and green solvents in the membrane technology because of their nontoxicity, low viscosity, low vapor pressure, prominent tunabilty, easy preparation, high biocompatibility and biodegradability [31]. It can be synthesized by mixing and heating at least two chemicals that are hydrogen bond donors (HBD) and hydrogen bond acceptors (HBA) [32]. Therefore, DES was synthesized by cetrimonium bromide (CTMB) and acetic acid as HBA and HBD, respectively. The nanoparticles (NPs) of ceria were synthesized by using cerium (IV) ammonium nitrate, ethylene glycol and isopropanol. DES was immobilized on ceria NPs by using ethanol via a solvent evaporation technique and filler for the MMMs was obtained. Polysulfone was used as support material to maintain the strength of the membrane matrix as it is easy to process, mechanically strong, and thermally and chemically stable. Finally, mixed matrix membranes of 2%, 4%, 6% and 8% DES immobilized ceria NP loadings were synthesized and analyzed by FTIR, SEM and TGA. Also, gas permeation was evaluated on the permeation of CO_2_ and the selectivities of CO_2_/N_2_ and CO_2_/CH_4_ for the detection of CO_2_ mitigation from the gaseous mixture. The results obtained proved that this combination tries to overcome and to optimize the trade-off between permeability and selectivity in the Robeson plot and lie near the line that might be a potential region of the plot. No doubt, it was not able to completely overcome the Robeson upper bound limit, but it still has potential improvement to be reported in comparison with pure polysulfone membranes.

## 2. Results and Discussion

### 2.1. Scanning Electron Microscopy (SEM)

**Figure 1:**
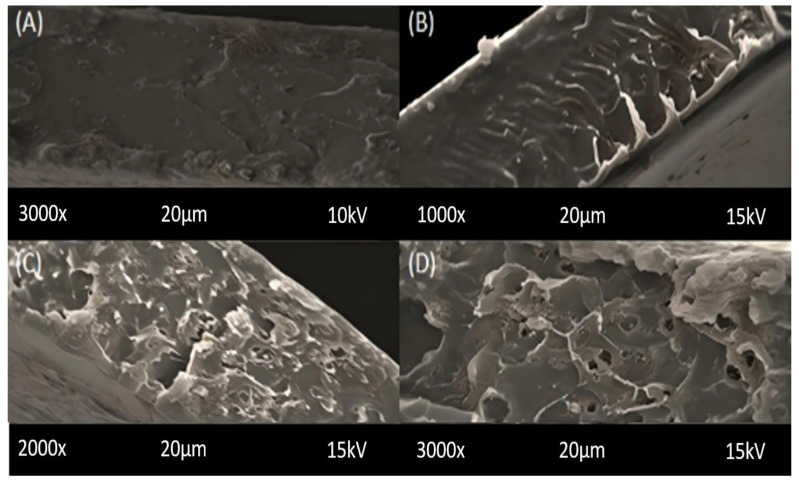
SEM images of cross-sections of MMMs of different filler loadings (**A**) 2% (**B**) 4% (**C**) 6% (**D**) 8%.

SEM (Tescan Vega (LMU)) was utilized to observe the cross-sectional and surface morphology of the membranes of different NP loadings (Figure 1 and Figure S1). Membranes were broken in liquid nitrogen to fix the cross-sectional ends of the membranes and a voltage of 15–20 Kv at different magnifications was applied with gold coating of the samples in the SEM machine. A SEM micrograph is used to observe the aggregation, dispersion and the existence of the filler NPs in the membrane matrix. SEM images show the increasing NP loadings as 2%, 4%, 6% and 8% in the membrane and the filler is highly dispersed across the whole membrane in each case. All of the membranes are accurately synthesized, and the structural density of the composition is visible without any imperfections. Some of the ripples can be seen with the increase of filler loadings that are due to inert NP aggregation that leads to agglomeration in greater than 8% NP loadings. Obviously, the nanoparticles form agglomerates due to the strong interaction between the modified small particles which can be a consequence of generating channels or voids that indicate the molecular chains’ flexibility in the membrane matrix [33]. The agglomeration is also due to the fact that the shape of the DES-modified ceria NPs cannot be assumed [34]. The main contribution of the selective gas permeation and membrane strength depends upon the pore structure of MMMs [35], which is obvious from the cross-section morphology. The springy asymmetric structures shown are due to the strong contact between the filler and polysulfone, and poor attraction of tetrahydrofuran (THF), which is the organic solvent that caused the skin-like structures while drying [36]. Hence the uniform and homogeneous dispersion of the filler exhibited by the images point towards the enhanced permeation of CO_2_ and the relative selectivities. The pore formation increases with the increase of DES-ceria which supports the hypothesis.

### 2.2. Fourier Transform Infrared (FTIR) Spectroscopy

**Figure 2:**
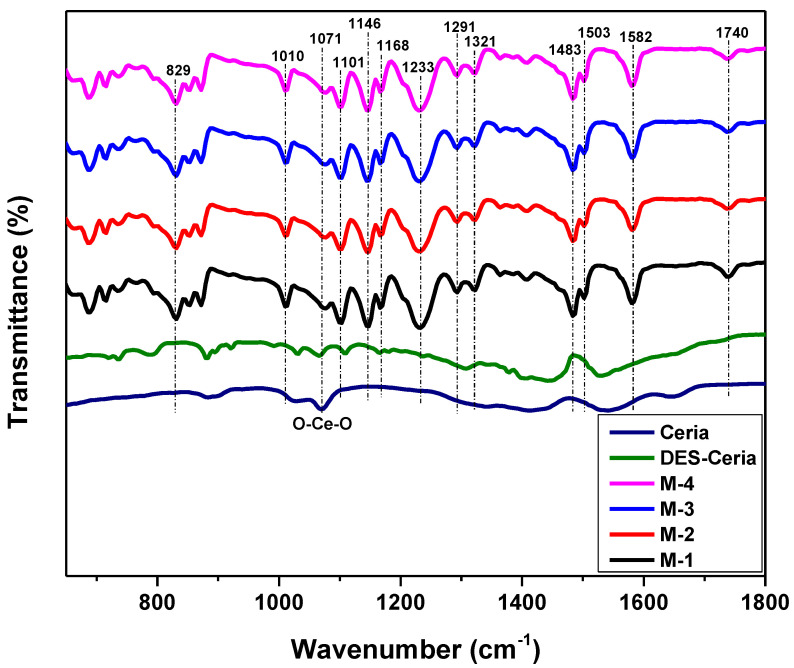
FTIR spectra for the ceria, DES-ceria and membrane compositions.

Figure 2 demonstrates the FTIR spectra for ceria, DES-immobilized ceria and all of the four compositions of the membranes in a 650–1800 cm^−1^ range. The major peak of ceria, that is the O-Ce-O stretching vibration, is at 1071 cm^−1^ [37,38,39]. It can be observed in all of the membrane compositions that indicated the presence of ceria filler in the synthesized MMMs. The peak at 1582 cm^−1^ indicates C=C stretching vibrations and benzene ring stretching [40]. The peaks at 1291 cm^−1^, 1321 cm^−1^ and 1483 cm^−1^ are the peaks of symmetric O=S=O stretching vibrations and C-H bending vibrations. The peaks at 1168 cm^−1^ shows C-O bending vibrations. The vibrational elongating symmetric behavior and asymmetric stretching vibrations of O=S=O bonds is represented by peaks at 1101 cm^−1^ and at 1146 cm^−1^, respectively, which is a clear indication that sulfone groups exist. The peak at 1233 cm^−1^ is a clear exhibition of the elongating vibration of Benz-O-Benz bonds, where Benz represents the benzene rings/aromatic functional group of PSF. The C=C stretching mode in aromatic compounds lies in the range 1485–1590 cm^−1^, which covers the peak at 1503 cm^−1^. The peak near 1710 cm^−1^, that is 1740 cm^−1^, indicates the symmetric stretching of a C=O bond, and at 829 cm^−1^ indicates the C_6_H_6_ ring bending. The peak at 1010 cm^−1^ represents C-H stretching of the C_6_H_6_ ring in PSF. The small shift in the peaks of the membrane in comparison with ceria NPs indicates the strong interface of the filler and polymer at the molecular level. A summary of all the peaks found can be seen in Table 1.

### 2.3. Thermogravimetric Analysis

**Figure 3:**
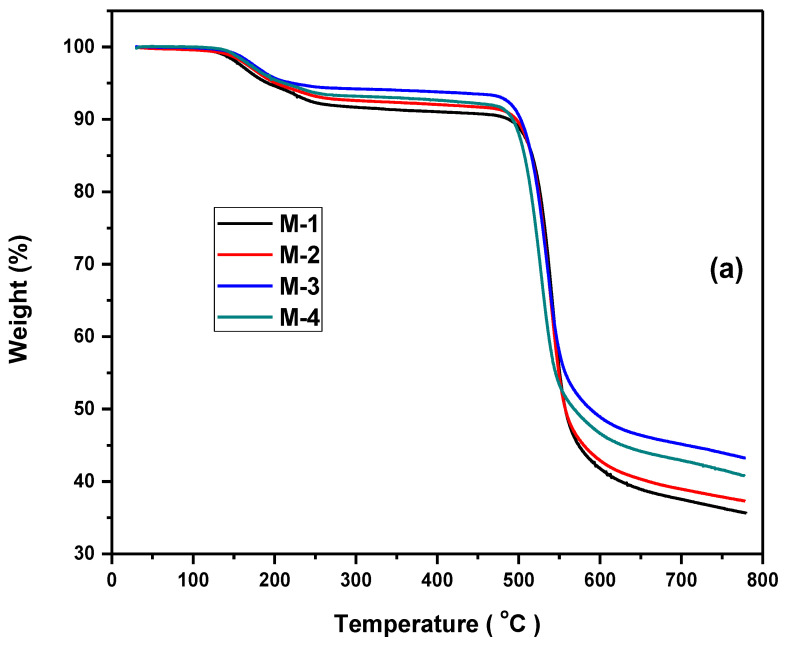

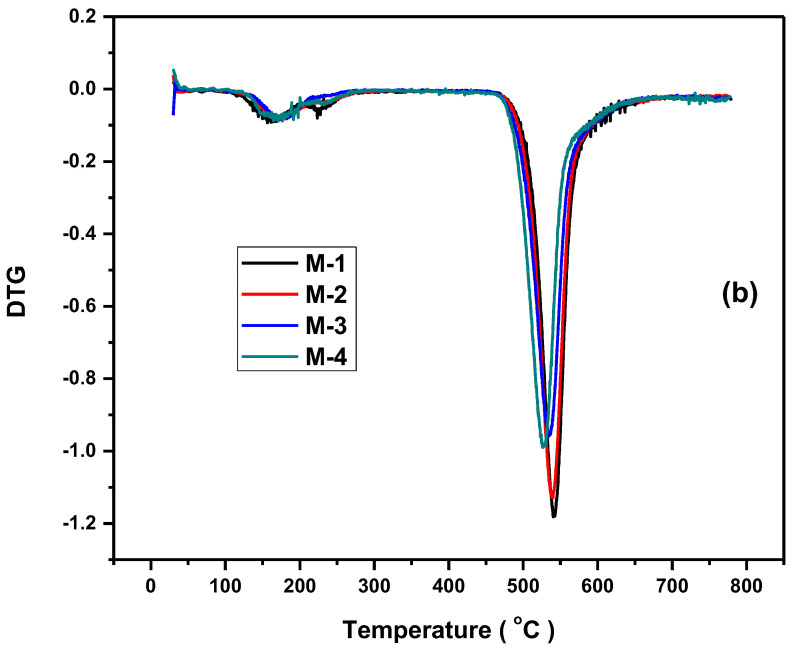
(**a**) Thermogravimetric analysis of membrane compositions. (**b**) Differential thermogravimetric analysis of membranes.

The thermogravimetric analysis was performed using a TGA analyzer (Perkin-Elmer STA 6000, Waltham, MA, USA), which is shown in Figure 3a, and the differential thermogravimetry is shown in Figure 3b to assess the thermal stability of MMMs. The MMMs were cut with a sample weight of 10 mg and placed in an oven at 100 °C to get rid of the enduring solvents and moisture, and then were cooled to room temperature. The cooled samples were placed in a sample holder and the temperature was adapted from 30 °C to 800 °C under a N_2_ environment with a 10 °C/min heating rate. The TGA is better than other isothermal conventional methods for the evaluation of thermal decomposition; this is because very small amounts of the sample are enough for the investigation. All of the MMMs were found to be stable up to approximately 500 °C, which is an indication of quite high stability and the complex behavior of the TGA curves indicate the presence of multiple aromatic rings. The initial flatness of the curves is an indication of the removal of residual solvents, like THF, used in the synthesis process. The first 6% loss in the range of 110–230 °C leads to the amputation of the molecules. The further 4% decomposition between 230 °C to 500 °C represents decomposition of DES-ceria NPs, as the variations in the curves started with the change of composition which represents the increase of thermal stability with the increase of DES-ceria NPs filler loading. This improvement of thermal stability proves the strong interaction between DES-ceria NPs and the polymer matrix. The sudden weight loss after 500 °C demonstrates breakage of the structure of major aromatic portions of PSF [33]. The normal temperature of flue gases is not more than 100 °C [30,41,42], hence it is right to say that MMMs are suitable for the capturing of CO_2_ from flue gases under operating temperatures. All of these weight losses can also be observed in the DTG curves. These results are similar to other already reported studies [29,43,44,45].

### 2.4. Gas Permeation Analysis

The permeation of gases was evaluated using a custom-built system for analyzing the permeabilities and selectivities of mixed and pure gases. Permeate and retentate compositions were estimated with the help of a gas chromatograph (Agilent 7890, Santa Clara, CA, USA) that has twin thermal conductivity detectors. The overall scheme of the gas infiltration process has been reported elsewhere, as well as the construction and working of the setup [46]. Metallic support was utilized to place the membranes and seal them. The inlet gas mixture flow rate was 1 L/min. For membrane testing, the feed temperature and pressure of the system was kept at 25 °C and 10 bar. All of the measurements were taken a minimum of three times and average values were calculated. The solution-diffusion model was utilized for the transport mechanism via MMMs.

**Figure 4:**
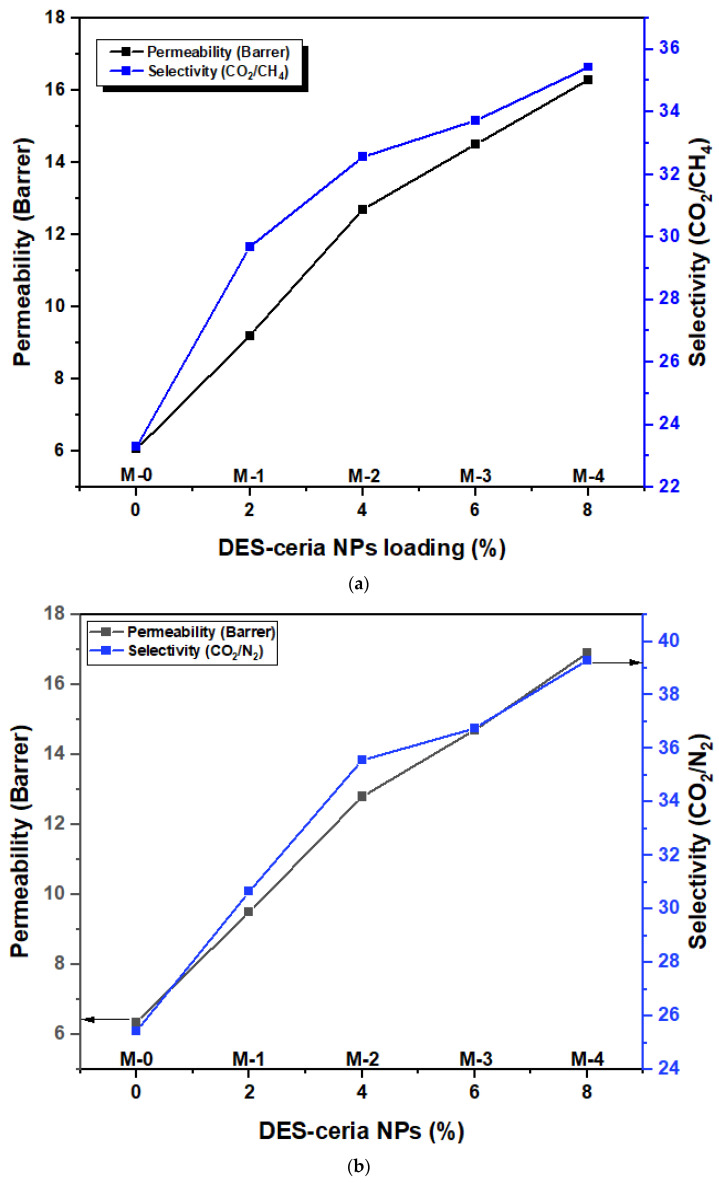
(**a**) Permeability and selectivity of MMMs for mixed gases CO_2_/CH_4._ (**b**) Permeability and selectivity of MMMs for mixed gases CO_2_/N_2_.

The permeabilities of CO_2_ and the selectivities (CO_2_/CH_4_ and CO_2_/N_2_) as a function of DES-ceria nanoparticle filler loadings are depicted in the Figure 4a,b. The variation in the permeabilities of different membrane compositions in comparison with pristine PSF membrane is termed as the facilitation ratio (FR). The FR is enhanced with the rise of filler from 2% to 4% DES-ceria NPs. The gas with smaller kinetic data (CO_2_ = 3.3Å) and a greater affinity with the amine and carboxyl groups present in DES exhibited an increased FR in comparison with gases of higher kinetic diameter (N_2_ = 3.64Å and CH_4_ = 3.8Å), which have a negligible affinity with the constituent functional groups of DES. This suggests that these results, within the same filler loading range, are due to the increment in pore density and the loss in pore size of membranes. The comparative study of the permeability of all three gases revealed the enhanced molecular sieving properties of DES-ceria NP-based MMMs that is the consequence of an overall increase in FR of CO_2_, N_2_ and CH_4_; this has a minor effect on the non-selective gases and gave better separation of the selective gas CO_2_. Hence, we conclude that the existence of DES-supported ceria NPs enhanced the molecular sieving properties of the membranes and improved the whole process of CO_2_ separation.

DES-ceria NP-based MMMs increased the CO_2_ permeability from 6.06 Barrer to 16.3 Barrer and the selectivity from 23.31 to 35.43 in the case of CO_2_/CH_4_ mixed gas. CO_2_ permeability increased from 6.36 Barrer to 16.9 Barrer and the selectivity from 25.44 to 39.3 in the case of CO_2_/N_2_ mixed gas. This is the FR of the gas permeation results. The difference between the results of the pure and mixed gases can be seen in Tables S1 and S2. This difference occurs because of the formation of the non-selective channels in MMMs. The incorporation of unambiguous functional groups was attempted to enhance CO_2_ separation from the mixed gases with MMMs, due to the affinity of the amine and carboxyl groups towards CO_2_. Hence, this combination was highly efficient at separation without any high selectivity loss.

**Figure 5:**
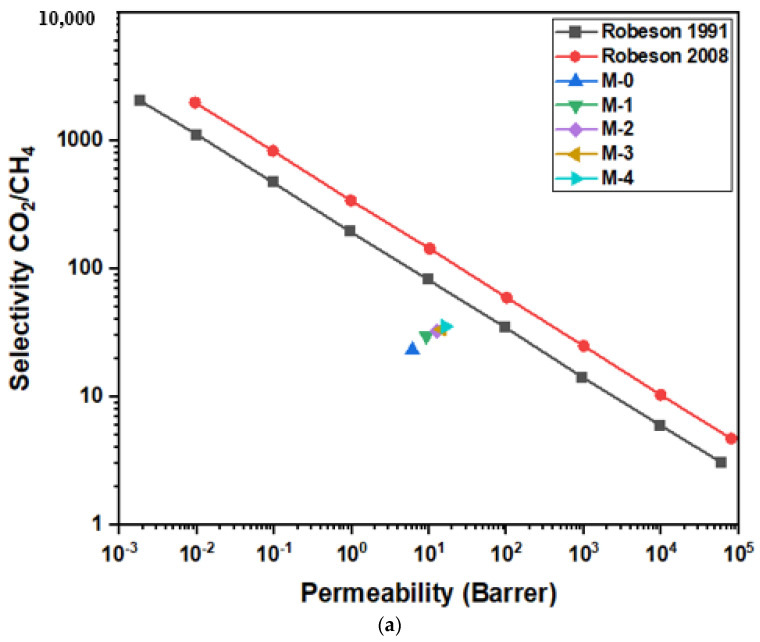

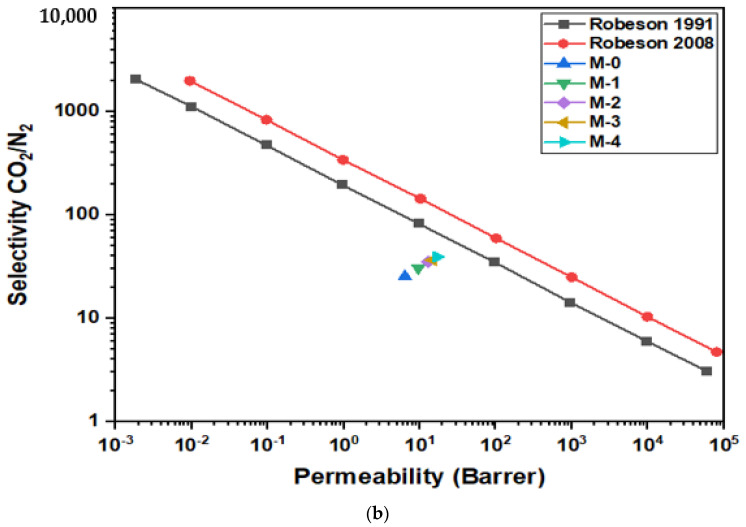
(**a**) Robeson plot comparison of MMMs for mixed gas CO_2_/CH_4._ (**b**) Robeson plot comparison of MMMs for mixed gas CO_2_/N_2_.

The results obtained from the permeation of gas analysis were calibrated with the famous Robeson plots as shown in the Figure 5a,b. The Robeson plot is an explanation of the association between permeability and selectivity, and it demonstrates the trade-off between these; if the permeability increases, the selectivity decreased; and if permeability decreases, the selectivity increases. The Robeson lines are considered the ideal lines for researchers to approach [14,29,47]. It is apparent that the points are moving towards the Robeson lines with respect to an increase in the filler loadings and the trade-off for the M-4 membrane is less than M-0. Therefore, in comparison with the pure PSF membrane, the 8% DES-ceria NP-based membrane has a smaller trade-off and lies in the technically strong region of the plot. Hence, the synthesized DES-ceria NP-based MMMs can be considered as potential candidates to enhance the efficiency of CO_2_ separation from gaseous mixtures [48].

## 3. Materials and Methods

### 3.1. Materials

PSF of A.M.W ~22,000, cetrimonium bromide (CTMB) (Purity ≥ 98%), and acetic acid (Purity ≥ 99%) were purchased from Sigma-Aldrich (St. Louis, MO, USA). Tetrahydrofuran (THF) (Purity = 99.6%), isopropyl alcohol (Purity ≥ 99.5%), ethylene glycol (Purity = 99%) and acetone (Purity ≥ 95%) for washing purposes were purchased from Fisher Scientific. Ammonium cerium (IV) nitrate (Purity = 99.5%) was obtained from Scharlau. A Water Purification Unit (Adrona SIA-B30, Riga, Latvia) is used to obtain Ultrapure water for washing purposes.

### 3.2. Deep Eutectic Solvent (DES) Preparation

**Figure 6:**
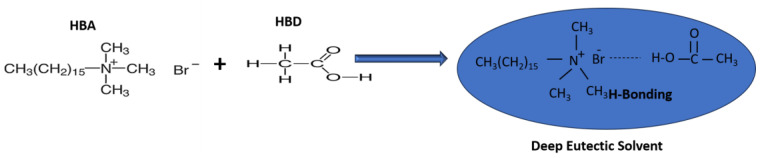
Synthesis of DES and H-bonding between CTMB and acetic acid [33].

The synthesis of DES was reported in our previous publication [33]. Briefly, DES was formed by combining equal masses of cetrimonium bromide (CTMB) and acetic acid at 70 °C for 3 h to obtain a homogeneous mixture. The synthesis was confirmed physically by analyzing the lowering of melting point as compared to the separate constituents and chemically by FTIR analysis. Figure 6 shows that CTMB was the hydrogen bond acceptor (HBA) and acetic acid was the hydrogen bond donor (HBD) [37].

### 3.3. Ceria Nanoparticles formation

The method of formation of the ceria nanoparticles has already been reported. Briefly, the uniform red-colored solution was formed by adding 0.3 g ammonium cerium (IV) nitrate into a mixture of 10 mL ethylene glycol and 10 mL isopropyl alcohol. The small amount of acetic acid (1 mL) was added to the above mixture dropwise with continuous stirring. The red solution was converted to a uniform colorless solution after 2 h of stirring. A Teflon-lined hydrothermal autoclave was used as a batch reactor and the resultant mixture was placed in an oven at 130 °C for 7 h. The yield was in the form of a greenish-brown mixture when cooled to room temperature. Ceria nanoparticles were obtained by centrifugation of the resultant mixture for 5 min at 8000 rpm. The solvents that were unreacted were recycled to avoid losses. The product was cleaned 3 times with ethanol and 1 time using acetone to achieve dried cerium oxide nanoparticles. After drying, 0.67 g of NPs was formed. In the recycling process, 0.3 g ammonium cerium (IV) nitrate was mixed with the unreacted solvents, then 0.7 mL acetic acid was added to the solution and the same procedure was repeated. The yield was 0.59 g in the first recycling process. In the second recycling process, the same procedure was repeated with 0.5 mL acetic acid and the yield was 0.50 g.

### 3.4. Surface Modification of Cerium Oxide Nanoparticles by DES

**Figure 7:**
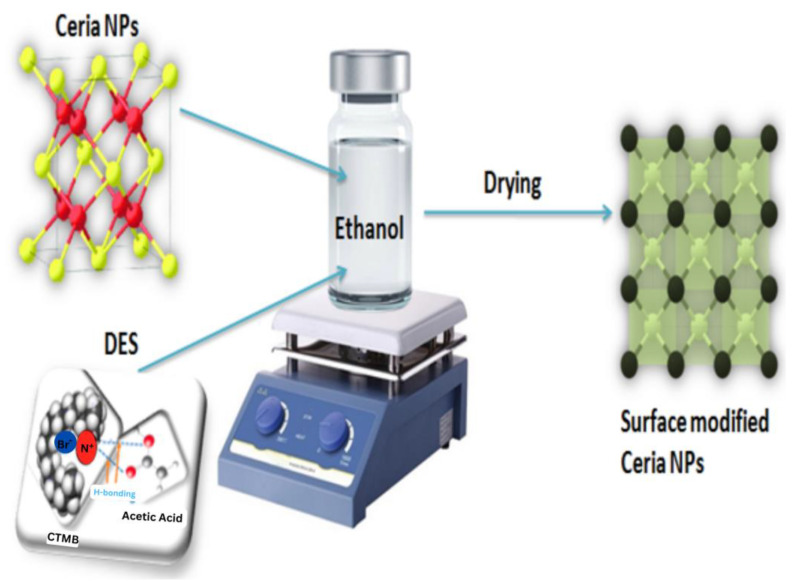
Surface tuning of ceria nanoparticles using CTMB-based deep eutectic solvent [33].

The DES-modified cerium oxide NPs were obtained by mixing the NPs with DES with a 8:1 ratio by weight into the small amount of evaporated organic solvent that is ethanol; the agglomeration was reduced using a mortar and pestle before using ethanol. The mixture was stirred to homogenize and placed at room temperature to evaporate the complete solvent for 24 h. The yield obtained after drying, that is the filler for MMMs, was named DES-ceria [33]. The work-flow of the surface tuning of ceria NPs is shown in Figure 7.

### 3.5. Mixed Matrix Membranes Fabrication

**Figure 8:**
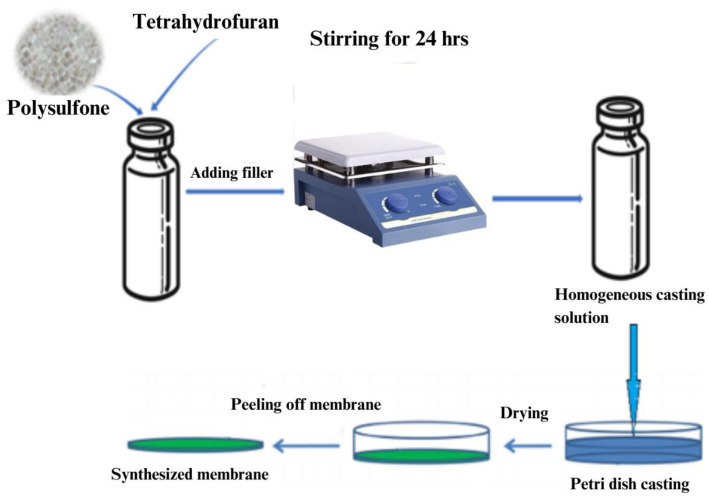
Schematic diagram for synthesis of mixed matrix dense membrane [33].

MMMs of various filler loadings were synthesized using DES-ceria nanoparticles as a filler with the help of a solution casting technique for dense membrane synthesis as shown in Table 2. PSF was mixed in THF for 2 h in a viol at room temperature with stirring of 500 rpm. After complete dissolution of the PSF, filler was added to the solution and mixed for 24 h under the same conditions to form a uniform composition. The air bubbles were removed using the gravity method and the solution was placed into a flat-bottom petri dish and left for 24 h to dry. After complete solvent evaporation, the membrane was peeled off from the bottom. The whole process of MMM synthesis is shown in Figure 8.

### 3.6. Characterization of Membrane Samples

The Tescan Vega LMU variable pressure Scanning Electron Microscope (Brno-Kohoutovice Czech Republic) was utilized to observe the cross-sectional and surface morphologies of the membrane samples. The Thermo Scientific Nicolet iS5 instrument from the United States was used to conduct the FTIR (Fourier Transform Infrared) spectroscopic analysis of membrane samples. A TGA analyzer (Perkin-Elmer STA 6000, Waltham, MA, USA), was utilized to examine the thermal stability of membrane samples. The composition of gases was evaluated with the help of a gas chromatograph (Agilent 7890, Santa Clara, CA, USA).

## 4. Conclusions

Ceria NPs were synthesized and combined with DES to fabricate a novel filler. MMMs of DES-ceria NPs were developed using a solvent evaporation technique. The SEM, FTIR and TGA of the membranes were performed. The SEM micrograph revealed the cross-sectional and surface morphologies of the synthesized MMMs, which described that the DES-ceria NPs were uniformly distributed over the polymer matrix and also making agglomerates because of the magnetic effects among the particles. The FTIR spectra indicated characteristic peaks of all of the constituents that suggests that no chemical reaction took place in the preparation of the membranes as all of the characteristic peaks can be easily indicated with respect to their functional groups. The TGA provided the comparison of thermal stabilities of the membrane and that the membrane with the highest filler loading was the most stable membrane which highlights the thermal stability of DES-ceria NPs, and that the overall trend for thermal stability was ascending from 0% to 8% DES-ceria NPs. The gas permeation analysis was carried out using pure and mixed gases and highlighted the enhanced permeability of CO_2_ and the selectivity as compared to a pristine PSF membrane. The improvement in the permeability of CO_2_ in pure and mixed gases (CO_2_/CH_4_ and CO_2_/N_2_) was from 6.7 Barrer to 17.2 Barrer, from 6.06 Barrer to 16.3 Barrer and from 6.36 Barrer to 16.9 Barrer, respectively. The improvement in the selectivities of the pure gases was from 24.81 to 41.95 (CO_2_/CH_4_) and from 26.8 to 40.95 (CO_2_/N_2_), and for the mixed gases was from 23.31 to 35.43 (CO_2_/CH_4_) and from 25.44 to 39.3 (CO_2_/N_2_). Thus, the DES-ceria NP-based MMMs enhanced the permeability as well as the selectivity and proved to be a promising filler for the mitigation of CO_2_ from a gaseous mixture. In future, mathematical modeling can also be applied by obtaining statistical data for different parameters relevant to the presented work, and also further modification of the nanoparticles by tunable DESs can open up a way for CO_2_ separation via MMMs.

## Figures and Tables

**Table 1:** Summary of FTIR spectra.

Sr No.	Wavenumber (cm^−1^)	Functional Groups
1	829	Benzene ring bending
2	1010	C-H stretching in benzene ring
3	1071	O-Ce-O stretching vibration
4	1101	Symmetric elongating vibration of O=S=O
5	1146	Asymmetric stretching vibration of O=S=O
6	1168	Bending vibration of C-O
7	1233	Elongating vibration of -C_6_H_4_-O-C_6_H_4_-
8	1291, 1321	Symmetric stretching vibration of O=S=O
9	1483	Bending vibration of C-H
10	1503, 1582	Stretching mode of C=C in aromatics
11	1740	Symmetric stretching of C=O

**Table 2:** MMMs composition by weight percentage.

Membrane Id	Membrane Type	PSF	Ceria	THF
M-0	PSF	4.8	-	95.2
M-1	2% ceria membrane	4.8	0.1	95.1
M-2	4% ceria membrane	4.8	0.2	95.0
M-3	6% ceria membrane	4.8	0.3	94.9
M-4	8% ceria membrane	4.8	0.4	94.8

## Data Availability

The data will be available on request.

## Supplementary Materials

The following supporting information can be downloaded at: https://www.mdpi.com/article/10.3390/molecules28207162/s1, Figure S1: Surface images of synthesized membranes; Table S1: The pure gas permeability and ideal gas selectivity of DES-ceria NPs based MMMs; Table S2: The mixed gas permeabilities and selectivities of DES-ceria NPs based MMMs.
