# Supplementary material for: Deep Eutectic Solvent Coated Cerium Oxide Nanoparticles Based Polysulfone Membrane to Mitigate Environmental Toxicology
Source: Molecules. 2023 Oct 19;28(20):7162. doi: 10.3390/molecules28207162 (PMC10609010; doi:10.3390/molecules28207162)
Supplement: Supplementary file 1 [file molecules-28-07162-s001.zip › molecules-2634216-supplementary.pdf]

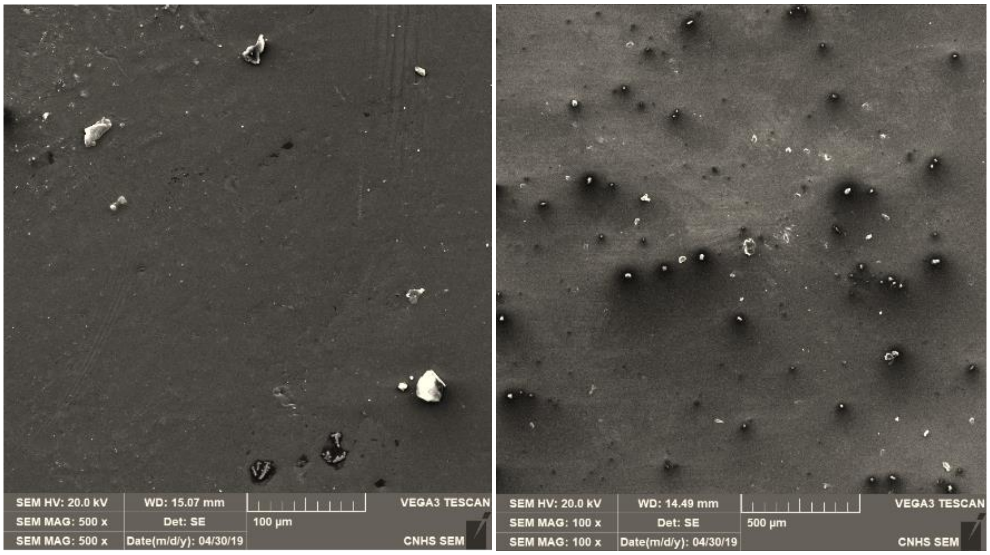

Figure S1: Surface images of synthesized membranes

Table S1: The pure gas permeability and ideal gas selectivity of DES-ceria NPs based MMMs

Membrane	Permeability (Barrer)			Selectivity	
	CO_2	CH_4	N_2	CO_2/CH_4	CO_2/N_2
M-0	6.7	0.27	0.25	24.81	26.8
M-1	9.9	0.32	0.3	30.94	33
M-2	13	0.37	0.35	35.14	37.14
M-3	14.9	0.39	0.38	38.21	39.21
M-4	17.2	0.41	0.42	41.95	40.95

Table S2: The mixed gas permeabilities and selectivities of DES-ceria NPs based MMMs

**Membrane**	**Permeability (Barrer)**		**Selectivity**	**Permeability (Barrer)**		**Selectivity**
	**CO_2**	**CH_4**	**CO_2/CH_4**	**CO_2**	**N_2**	**CO_2/N_2**
M-0	6.06	0.26	23.31	6.36	0.25	25.44
M-1	9.2	0.31	29.68	9.5	0.31	30.65
M-2	12.7	0.39	32.56	12.8	0.36	35.56
M-3	14.5	0.43	33.72	14.7	0.4	36.75
M-4	16.3	0.46	35.43	16.9	0.43	39.3
